# A Strategy of NIR Dual‐Excitation Upconversion for Ratiometric Intracellular Detection

**DOI:** 10.1002/advs.201901874

**Published:** 2019-09-24

**Authors:** Jianxi Ke, Shan Lu, Xiaoying Shang, Yan Liu, Hanhan Guo, Wenwu You, Xingjun Li, Jin Xu, Renfu Li, Zhuo Chen, Xueyuan Chen

**Affiliations:** ^1^ CAS Key Laboratory of Design and Assembly of Functional Nanostructures State Key Laboratory of Structural Chemistry and Fujian Key Laboratory of Nanomaterials Fujian Institute of Research on the Structure of Matter Chinese Academy of Sciences Fuzhou Fujian 350002 China; ^2^ School of Physical Science and Technology ShanghaiTech University Shanghai 201210 China; ^3^ University of Chinese Academy of Sciences Beijing 100049 China

**Keywords:** dual excitation, dye sensitization, intracellular detection, ratiometric probes, upconversion nanoparticles

## Abstract

Intracellular detection is highly desirable for biological research and clinical diagnosis, yet its quantitative analysis with noninvasivity, sensitivity, and accuracy remains challenging. Herein, a near‐infrared (NIR) dual‐excitation strategy is reported for ratiometric intracellular detection through the design of dye‐sensitized upconversion probes and employment of a purpose‐built NIR dual‐laser confocal microscope. NIR dye IR808, a recognizer of intracellular analyte hypochlorite, is introduced as energy donor and Yb,Er‐doped NaGdF_4_ upconversion nanoparticles are adopted as energy acceptor in the as‐designed nanoprobes. The efficient analyte‐dependent energy transfer and low background luminescence endow the nanoprobes with ultrahigh sensitivity. In addition, with the nonanalyte‐dependent upconversion luminescence (UCL) excited by 980 nm as a self‐calibrated signal, the interference from environmental fluctuation can be alleviated. Furthermore, the dual 808/980 nm excited ratiometric UCL is demonstrated for the quantification of the level of intracellular hypochlorite. Particularly, the intrinsic hypochlorite with only nanomolar concentration in live MCF‐7 cells in the absence of exogenous stimuli is determined. Such an NIR dual‐excitation ratiometric strategy based on dye‐sensitized UCL probes can be easily extended to detect various intracellular analytes through tailoring the reactive NIR dyes, which provides a promising tool for probing biochemical processes in live cells and diagnosing diseases.

## Graphical abstract

Near‐infrared (NIR) dye‐sensitized upconversion nanoprobes are designed for intracellular detection with high sensitivity and accuracy via a purpose‐built NIR dual‐laser confocal microscope system, providing a useful tool to study biological activities in live cells.

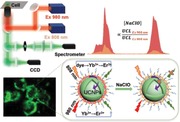

## 1 Introduction

Noninvasive luminescent monitoring of biological molecules or physicochemical parameters in live cells represents a key approach to understanding cell biology, pathology, and other biomedical‐related sciences.^1^ However, current intracellular analyses by means of target imaging or nonquantitative fluorescence (FL) contrast are still far from satisfactory.^1^, ^2^ The main obstacle is the inadequate sensitivity of the probes to the target species. For instance, the intrinsic intracellular hypochlorite in cancer cells without exogenous stimuli was undetectable in most cases due to its extremely low concentration.[qv: 2b,f] Although some dye probes exhibited ultralow detection limit from in vitro assays, both attributes of poor water solubility and short‐wavelength excitation hindered their extensive utilization in a live‐cell context.^3^ In addition, accurate quantitative detection based on these dyes was hardly achieved without a self‐calibrated reference.^4^ The detection deviations in live cells may derive from the uncertain factors of complex intracellular interferents, inhomogenous probe distribution as well as instrumental set‐ups.[qv: 4a,5]

As a new generation of luminescent bioprobes, lanthanide‐doped upconversion nanoparticles (UCNPs) feature near‐infrared (NIR) excitation and exhibit unique advantages such as low toxicity, little photodamage, low autofluorescence, and deep light penetration in biological specimens.^6^ During the past decade, UCNPs‐based nanoprobes have been extensively developed to detect various intracellular ions, small molecules, or macromolecules.^7^ Such biodetections are generally achieved by monitoring luminescence resonance energy transfer (ET) between UCNPs donor and energy acceptor (like organic dyes). Therefore, the detection sensitivity depends critically on both the upconversion luminescence (UCL) intensity and the quenching efficiency. Normally, UCNPs with larger size or core–shell structure emit stronger UCL but their inside activator ions cannot be efficiently quenched by surface‐bound acceptors.[qv: 7h] Alternatively, NIR dyes as antennas for UCNPs sensitization can significantly enhance the UCL by increasing the absorption cross‐sections and optical bandwidths.^8^ In one of the pioneering works, a total of 3300‐fold increase in overall upconversion emission of NaYF_4_:Yb,Er was achieved through dye sensitization upon excitation at 720–1000 nm.^9^ Such efficient ET from organic dyes to UCNPs provides a new approach to sensitive biodetection.^10^ Despite the great potential of dye‐sensitized UCL probes, their utilization in intracellular detection has been rarely explored. Recently, a nanoprobe comprised of NIR dyes and NaYF_4_:Yb,Er@NaYF_4_:Nd UCNPs was demonstrated to monitor the alteration of GSH level in live HeLa cells.[qv: 10a] Through dye sensitization, the nanoprobe afforded the maximal signal‐to‐background ratio (S/B) of 30, which is about threefolds of most existing dye‐quenched (or other acceptors) UCL nanoprobes. However, this value is still far below the anticipated S/B for dye‐sensitized UCNPs according to the maximal UCL enhancement factor. Such a difference may be due mainly to their close excitation wavelengths at ≈800 nm between Nd^3+^ and NIR dye, which results in an inevitable high background interference. Furthermore, ratiometric quantitative detection in live cells by employing the dye‐sensitized UCL probes has not been attempted. In particular, dual‐emission ratiometric detection strategy generally adopted for dye‐quenched UCL probes might not suite dye‐sensitized UCL probes in view of simultaneous change of all UCL emissions.

**Figure 1:**
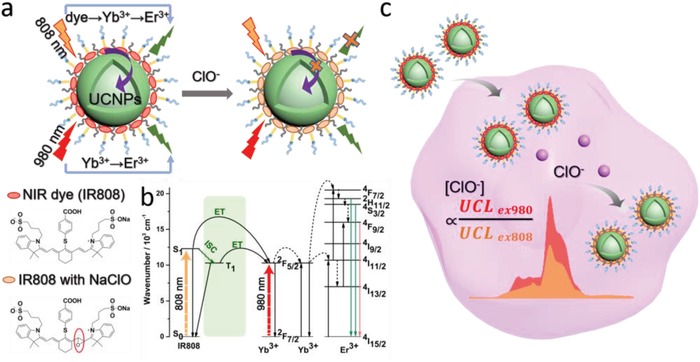
Schematic illustration of analyte‐dependent ET in NIR dye‐sensitized upconversion nanoprobes. a) The composition of 980/808 nm dual‐excitation nanoprobes and the mechanism of dye‐sensitized UCL quenching by ClO^−^. b) ET landscape, showing intersystem crossing of NIR dye from the singlet (S_1_) to the triplet state (T_1_) before transfer to UCNP lanthanides. c) Ratiometric detection of intracellular ClO^−^ with the as‐designed nanoprobes.

To meet the demand for intracellular detection with high sensitivity and accuracy, we propose a strategy of NIR dual‐excitation ratiometric UCL detection based on dye‐sensitized nanoprobes overcoming the limitation aforementioned via a purpose‐built NIR dual‐laser confocal microscope. Intracellular hypochlorite (ClO^−^), one of the most important reactive oxygen species (ROS) associated with the immune diseases, is chosen as the model analyte in this work.[qv: 7c] As shown in **Figure** **1**, the nanoprobes are composed of ClO^−^‐recognizer NIR cyanine dye IR808 as energy donor, and core–shell Yb,Er‐doped UCNPs as energy acceptor. The efficient analyte‐dependent ET and the large absorption discrepancy at 808 nm between NIR dye and lanthanide ions (Yb^3+^ and Er^3+^) endow the nanoprobes with ultrahigh detection sensitivity. Furthermore, UCL spectra under 808 and 980 nm excitation can be separately corrected at a fixed position of cell by the developed microscope system. The UCL excited by 980 nm is independent of the analyte concentration and able to alleviate the interference of intracellular fluctuation. With this ratiometric measurement, more accurate quantification of intrinsic and exogenous ClO^−^ in live cancer cells could be achieved. To the best of our knowledge, such unique design strategy based on the NIR dual‐excitation ratiometric UCL for intracellular detection had never been reported before.

## 2 Results and Discussion

**Figure 2:**
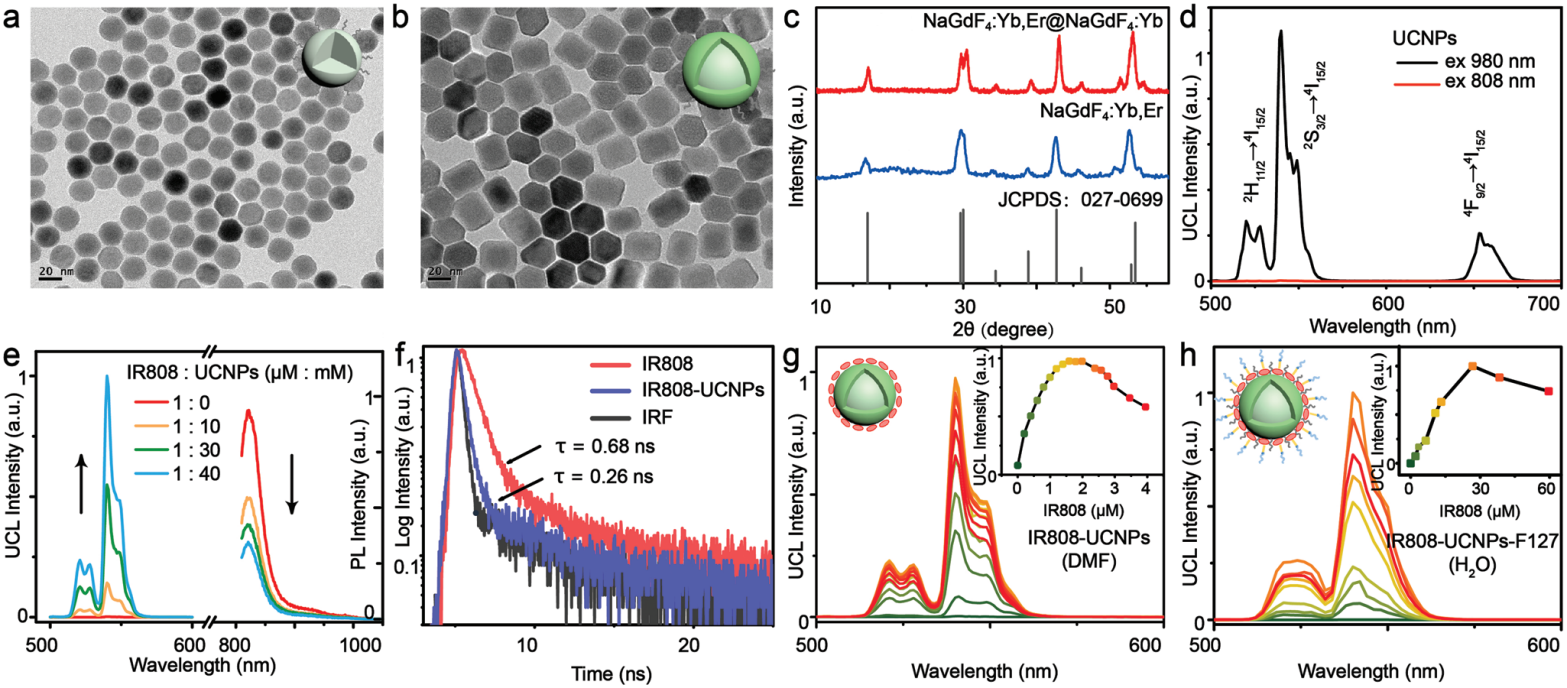
TEM images of a) NaGdF_4_:Yb,Er core and b) NaGdF_4_:Yb,Er@NaGdF_4_:Yb core–shell UCNPs. c) XRD patterns of the core and core–shell UCNPs. d) UCL spectra of core–shell UCNPs in DMF (33 × 10^−3^ m) under 980 or 808 nm excitation with a power density of ≈2.2 W cm^−2^. e) UCL spectra and emission spectra of IR808 anchoring to different amount of UCNPs in DMF under 808 nm excitation. f) FL lifetime of the IR808 in the absence and the presence of UCNPs (UCNPs: 33 × 10^−3^ m, IR808: 2 × 10^−6^ m). UCL spectra of g) IR808‐UCNPs in DMF and h) IR808‐UCNPs‐F127 in water with different IR808 concentrations under 808 nm excitation (UCNPs: 33 × 10^−3^ m). Inset: Integrated UCL intensity (500–600 nm) as a function of the IR808 concentration.

We employed NaGdF_4_:Yb,Er@NaGdF_4_:Yb as UCNPs in view of the energy overlap between the excited T_1_ state of NIR cyanine dye and the Yb^3+^ ground‐state absorption, and the successive dye→Yb^3+^→Er^3+^ ET process may realize highly efficient UCL (Figure 1b).^11^ The oleic acid (OA)‐capped core–shell UCNPs were prepared through a facile solid‐liquid‐thermal‐decomposition (SLTD) method previously reported.^12^ Transmission electron microscopy (TEM, **Figure** **2**a,b and Figure S1, Supporting Information) images and X‐ray diffraction (XRD, Figure 2c) patterns showed that NaGdF_4_:18%Yb,2%Er core‐only and NaGdF_4_:18%Yb,2%Er@NaGdF_4_:20%Yb core–shell nanoparticles are hexagonal (JCPDS No. 027‐0699) and uniform, with a mean size of 24.6 and 29.0 nm, respectively (Figure S1, Supporting Information). NIR dye IR808 was synthesized by three steps according to Scheme S1 in the Supporting Information and further verified by NMR and mass spectrometry (Figures S2–S4, Supporting Information). As the organic antenna, IR808 possesses carboxyl group for anchoring to the UCNPs surface, which guarantees an efficient ET between IR808 and UCNPs.^9^, ^13^

Under 980 nm laser excitation, the core–shell UCNPs exhibited characteristic UCL peaks, which were attributed to the ^2^H_11/2_→^4^I_15/2_ (521 nm), ^4^S_3/2_→^4^I_15/2_ (541 nm), and ^4^F_9/2_→^4^I_15/2_ (654 nm) transitions of Er^3+^ by energy absorption from Yb^3+^ (Figure 2d). In sharp contrast, the UCL emission was almost negligible under 808 nm irradiation with similar power density as 980 nm laser. When introducing IR808 to the surface of ligand‐free UCNPs in *N*,*N*‐dimethylformamide (DMF), the obtained composites (IR808‐UCNPs) showed visibly enhanced UCL through dye sensitization (Figure 2g). The UCL intensity under 808 nm excitation increased initially due to the enhanced overall absorption of the excitation energy via the participation of more antenna dyes (0 × 10^−6^–1.5 × 10^−6^ m), then declined caused by FL quenching with further increasing dye concentration (>1.5 × 10^−6^ m).^14^ In addition, the UCL and dye emission of IR808 anchoring to different amount of UCNPs were compared in Figure 2e. The gradual increase in UCL emission at the expense of dye emission provided strong evidence for the ET from IR808 dye to UCNPs. The FL lifetime of IR808 in the absence and in the presence of UCNPs was calculated to be 0.68 and 0.26 ns, respectively, from which the efficiency for ET was thus estimated to be 61.8% (Figure 2f).^15^

In order to further overcome the decreased absorption and FL intensity of IR808 and partial dissociation of IR808 from UCNPs in biological aqueous phase,[qv: 14a] amphiphilic triblock copolymer Pluronic F127 was employed to wrap the hydrophobic dye‐sensitized UCNPs for the realization of sensitization and dispersion in water.[qv: 8b] TEM image indicated that the as‐prepared nanoprobes (IR808‐UCNPs‐F127) were well dispersed in water (Figure S5, Supporting Information). Fourier transform infrared (FTIR) spectroscopy confirmed the successful capping of IR808 and F127 on the surface of UCNPs (Figure S6, Supporting Information). The amount of loaded IR808 was measured by means of UV‐vis absorption spectra (Figure S7, Supporting Information). Upon assembly with F127, the sensitization effect of IR808 on UCNPs remained efficient in aqueous solution, which also showed typical dependence on the dye concentration (Figure 2h). Due to the protection of OA‐F127, the optimal dye concentration for IR808‐UCNPs‐F127 reached about 27 × 10^−6^ m, higher than that of IR808‐UCNPs. The optimal number ratios of IR808/UCNPs in IR808‐UCNPs and IR808‐UCNPs‐F127 were calculated to be 5:1 and 90:1, respectively. In order to achieve efficient ET, and more importantly, to ensure the positive correlation between UCL and dye (in consequence, the negative correlation between UCL and analyte), a proper dye concentration of 13.2 × 10^−6^ m (≈0.24% wt) was finally adopted in the nanoprobes for subsequent detection protocols.

**Figure 3:**
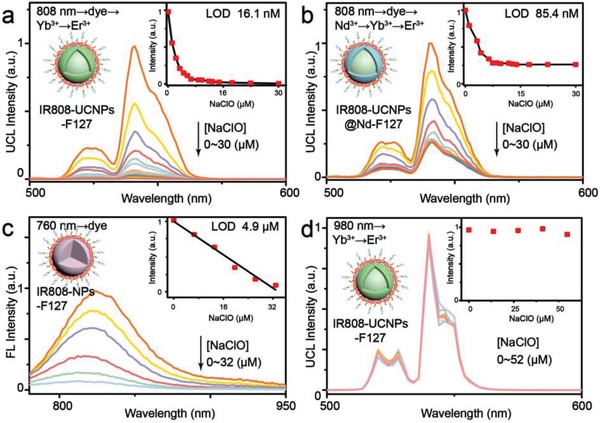
UCL spectra of IR808‐UCNPs‐F127 nanoprobes in water upon gradual addition of NaClO under excitation a) at 808 nm or d) 980 nm. b) UCL spectra of IR808‐UCNPs@Nd‐F127 in water upon gradual addition of NaClO under excitation at 808 nm. c) FL spectra of IR808‐NPs‐F127 in water upon gradual addition of NaClO under excitation of xenon lamp at 760 nm. Inset: Integrated UCL or FL intensity as a function of the NaClO concentration.

As one kind of heptamethine cyanine dyes, IR808 can be specifically oxidized by ClO^−^ under physiological conditions.^16^ Once ClO^−^ in the environment reacts with NIR dyes, the UCL sensitization pathway is interrupted, resulting in the quenching of dye‐sensitized UCL. As shown in **Figure** **3**a, the UCL intensity of IR808‐UCNPs‐F127 under 808 nm excitation was sharply decreased upon gradual addition of NaClO. Specifically, the UCL intensity was found to decrease approximately 178‐folds with the titration of 30 × 10^−6^ m NaClO, indicative of the maximal S/B as high as 178. The detection curve for the ClO^−^ concentration exhibits a linear dependence in the range of 0 × 10^−6^–3.2 × 10^−6^ m. The limit of detection (LOD), defined as the concentration that corresponds to three times the standard deviation above the signal measured in the blank, was determined to be 16.1 × 10^−9^ m.[qv: 7c] For comparison, IR808‐UCNPs@Nd‐F127 counterparts with the use of Nd/Yb/Er co‐doped UCNPs as energy acceptor were prepared and applied for detection under the same condition. The total UCL decrease of IR808‐UCNPs@Nd‐F127 was only 3.8‐folds upon NaClO addition and a higher LOD of 85.4 × 10^−9^ m was obtained (Figure 3b). Meanwhile, the detection using FL emission of NIR dyes was also analyzed. We synthesized optically inert NaGdF_4_ NPs to take place of UCNPs and prepared IR808‐NPs‐F127 counterparts without ET between IR808 and NPs. As observed in Figure 3c, the emission of IR808‐NPs‐F127 at 780–950 nm steadily decreased with the increasing amount of NaClO under excitation at 760 nm. The LOD was determined to be 4.9 × 10^−6^ m, which is much higher than that of IR808‐UCNPs‐F127. The designed IR808‐UCNPs‐F127 nanoprobes also outperform the previously reported dye‐quenched UCL probes that exhibited LOD of 0.5 × 10^−6^ and 0.32 × 10^−6^ m with energy acceptor of Cy3 and rhodamine, respectively.[qv: 7c,17] Moreover, under 980 nm laser irradiation, addition of NaClO (0 × 10^−6^–52 × 10^−6^ m) caused almost no effect on the UCL intensity of IR808‐UCNPs‐F127 (Figure 3d). As such, the UCL induced by direct excitation at 980 nm may establish an internal standard for ratiometric measurement.[qv: 4a]

To demonstrate the validity of nanoprobes in biological media, the anti‐interference ability from pH variations and other bioactive molecules was surveyed. It was observed that the UCL of IR808‐UCNPs‐F127 was basically stable at physiological pH ranging from 5.5 to 8.0 (Figure S8, Supporting Information). Importantly, the difference of UCL intensity in media between phosphate‐buffered saline (PBS) of pH 7.4 and the cell lysates of human embryo lung fibroblasts (HELF) cells was only ≈1%, suggesting that the nanoprobes are insensitive to the ordinary intracellular substances. In addition, UCL response was highly specific toward NaClO from various competing ROS, including hydrogen peroxide (H_2_O_2_), superoxide (·O^2−^), hydroxyl radical (·OH), and nitric oxide (NO) (Figure S9, Supporting Information). The specific reaction of IR808 with NaClO lies in the oxidation of the double bonds in the secondary amine substitution as shown in Scheme S2 in the Supporting Information.^16^ From ^1^H NMR spectroscopy (Figure S10, Supporting Information), the signals (3.4–3.7 ppm) of the C=C double bond in the IR808 were greatly reduced with the treatment of NaClO, which is consistent with the reaction mechanism. Subsequently, the cytotoxicity of IR808‐UCNPs‐F127 was evaluated according to the standard methyl thiazolyl tetrazolium assay. After incubation with 0–500 µg mL^−1^ nanoprobes for 12 or 24 h, all the viabilities of human breast cancer cell line (MCF‐7) remained above 94% (Figure S11, Supporting Information), indicating that the nanoprobes are essentially nontoxic to live cells. The UCL images of confocal microscopy excited at 980 nm prove the successful cellular uptake of nanoprobes (Figure S12, Supporting Information), and such a negligibly low cytotoxicity of nanoprobes is essential to avoid perturbing native cellular conditions.

**Figure 4:**
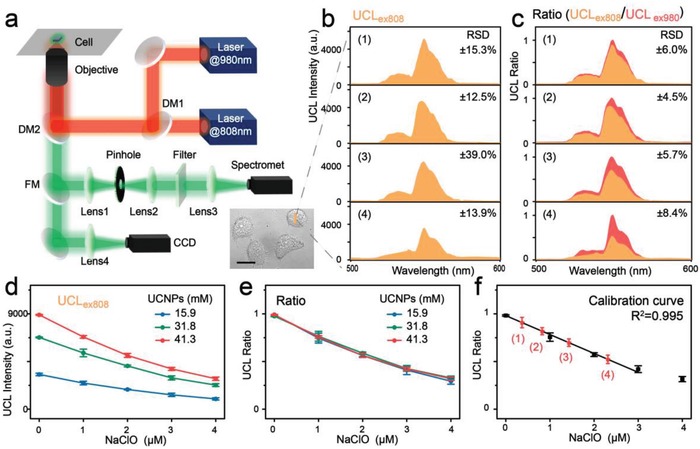
a) Optical layout of the purpose‐built NIR dual laser confocal microscope system (DM: dichroic mirror, FM: flip mirror, CCD: charge coupled device). b) UCL spectra collected at 500–600 nm under 808 nm excitation (UCL_ex808_) in probe‐loaded cells. Panels (1)–(4) showed the different cell groups upon addition of NaClO with 0 × 10^−6^, 0.5 × 10^−6^, 1× 10^−6^, or 2 × 10^−6^ m, respectively. c) UCL ratio (UCL_ex808_/UCL_ex980_) derived from (b) by normalizing the UCL intensities to their corresponding 540 nm emission under 980 nm excitation. RSDs of UCL_ex808_ and UCL ratio were given in (b) and (c), respectively (*n* = 6 cells). d) Plots of UCL_ex808_ versus the NaClO concentration. e) Plots of UCL ratio versus the NaClO concentration. f) Calibration curve established for NaClO quantification in live cells. The black dots represent the average UCL ratios of different nanoprobe concentration obtained from (e) for establishment of calibration curve. The red dots (1)–(4) represent the average UCL ratio in MCF‐7 cells pre‐treated by 0 × 10^−6^, 0.5 × 10^−6^, 1 × 10^−6^, and 2 × 10^−6^ m of NaClO, respectively, for determination of intracellular ClO^−^.

To perform the ratiometric intracellular detection, an NIR dual‐laser confocal microscope system was developed. As illustrated in **Figure** **4**a, the confocal laser microscope was equipped with two independent excitation sources, 980 and 808 nm continuous‐wave laser diodes. An oil‐immersion objective lens was used to focus the excitation light onto the sample cell. The UCL signal from the cells was collected by the same objective lens, reflected by a dichroic mirror and a flip mirror, and further detected by a spectrometer to acquire the full spectrum or projected onto a charged coupled device (CCD) camera to acquire images. By mean of the designed system, the dye‐sensitized UCL under 808 nm laser excitation (UCL_ex808_) and the UCL signal under 980 nm laser excitation (UCL_ex980_) can be separately captured from the same position of cell. The tested adherent cells have only several micrometers in height, therefore, the distinction of light attenuation in the cells between 808 and 980 nm is negligible. Each UCL spectrum was acquired within 100 ms to avoid dye bleaching caused by long‐time irradiation. In the photostability test, a decline of less than 5% in the UCL intensity was observed after continuous five spectral measurements (0.5 s) under 808 nm laser irradiation with a power density of ≈6 × 10^4^ W cm^−2^, indicative of the reliability of our test results (Figure S13, Supporting Information).

Recent studies have suggested that cancer cells exhibit higher ClO^−^ level than normal cells, yet the intrinsic ClO^−^ in cancer cells is at the nanomolar level and hard to be quantified.[qv: 2d] Thanks to ultrasensitivity and self‐calibration features of IR808‐UCNPs‐F127, we explored the nanoprobes to detect the intrinsic ClO^−^ in live MCF‐7 cells without exogenous stimuli via the NIR dual‐laser confocal microscope. In addition, the exogenous ClO^−^ with different levels in MCF‐7 cells were also assessed. Specifically, four groups of probe‐loaded MCF‐7 cells were treated with different concentration of NaClO (0 × 10^−6^, 0.5 × 10^−6^, 1 × 10^−6^ , and 2 × 10^−6^ m), respectively. The cells were washed and subjected to UCL measurement on the microscope. With the assistance of CCD camera, intracellular UCL spectra at 500–600 nm under 808 or 980 nm excitation for different cells were recorded. It was observed that the intensity of UCL_ex808_ from the different cells of the same group has very high deviations and the maximum of relative standard deviations (RSDs) reached 39.0% (Figure 4b). Such high deviations arose mainly from the fluctuation in probe distribution and intracellular environment. As the UCL intensities were normalized to the corresponding 540 nm emission under 980 nm excitation, the RSDs of UCL ratio (UCL_ex808_/UCL_ex980_) were reduced down to 4.5–8.4% (Figure 4c), illustrating that the strategy of dual‐excitation ratiometric UCL can effectively alleviate the environmental interference. The maximal S/B was further calculated to be 41.0 according to the ratiometric UCL. For quantification of ClO^−^ in the cells, the calibration curve was further established under the same condition such as laser intensity, focus, and irradiation time in the microscope system. As shown in Figure 4d,e, with the nanoprobe concentration varying from 15.9 × 10^−3^, 31.8 × 10^−3^, to 41.3 × 10^−3^ m, the plots of UCL_ex808_ versus the NaClO concentration also varied accordingly, while the plots of UCL ratio versus the NaClO concentration were basically identical with each other. Therefore, a calibration curve based on the UCL ratios was constructed for ClO^−^ detection with a linear range of 0 × 10^−6^–3.0 × 10^−6^ m (*R* ^2^ > 0.996) (Figure 4f). On the basis of the calibration curve, the intrinsic ClO^−^ in MCF‐7 cells was determined to be 0.28 ± 0.10 × 10^−6^ m, and the total intracellular accumulation of ClO^−^ in MCF‐7 cells pre‐treated by 0.5 × 10^−6^, 1 × 10^−6^, and 2 × 10^−6^ m of NaClO was determined as 0.82 ± 0.07 × 10^−6^ m, 1.43 ± 0.14 × 10^−6^ m, and 2.31 ± 0.25 × 10^−6^ m, respectively. The exogenous ClO^−^ can be estimated by subtracting the intrinsic ClO^−^ from the total value detected, which was close to the externally added amount. All these results demonstrate that our designed nanoprobes under NIR dual‐excitation ratiometric detection model can provide a feasible platform for intracellular detection with merits of specificity, ultrasensitivity, and high accuracy.

## 3 Conclusion

In summary, we have designed IR808‐UCNPs‐F127 nanoprobes for intracellular detection based on the analyte‐dependent ET from NIR dye to Yb,Er‐doped UCNPs. Benefiting from the efficient dye‐sensitization and low background luminescence, the nanoprobes exhibited ultrasensitive detection for ClO^−^ with an LOD of 16.1 × 10^−9^ m, which is much lower than that of IR808‐UCNPs@Nd‐F127 (85.4 × 10^−9^ m), IR808‐NPs‐F127 (4.9 × 10^−6^ m), or conventional dye‐quenched UCL probes. By means of a purpose‐built NIR dual‐laser confocal microscope, we have successfully realized dual‐excitation ratiometric UCL detection in live cells. Both intrinsic and exogenous ClO^−^ in MCF‐7 cells have been accurately quantified owing to the minimal interference from other biologically relevant analytes, pH perturbation, and inhomogenous probe distribution. Through modulating NIR dyes with other reactive groups, IR808‐UCNPs‐F127 can be extended to detect other intracellular analytes. These findings pave the way for designing ultrasensitive dye‐sensitized UCL probes and provide a general strategy of NIR dual‐excitation upconversion for ratiometric intracellular detection.

## 4 Experimental Section

*Chemicals*: Rare‐earth acetates, 1‐octadecene (ODE, 90%), OA (90%), Pluronic F‐127 (F127), and *N*‐[(3‐(anilinomethylene)‐2‐chloro‐1‐cyclohexen‐1‐yl)methylene] aniline hydrochloride were purchased from Sigma‐Aldrich. Sodium hypochlorite solid (available chlorine 42.2%) was purchased from TCI. 2,3,3‐trimethylindolenine, 1,4‐butanesultone, and deuterated reagent were purchased from Adamas‐beta Ltd. NaHF_2_, cyclohexane, ethanol, DMF, methanol, dichloromethane, diethyl ether, and 1,2‐dichlorobenzene (o‐DCB) were purchased from Sinopharm Chemical Reagent Co. Deionized water with resistivity of 18.2 MΩ cm was used in all experiments.

*Apparatus*: ^1^H and ^13^C NMR spectra were recorded on a Brucker spectrometer at 400 MHz. All chemical shifts were reported in the standard δ notation of parts per million. Mass spectra (MS) were obtained on a Bruker Impact II UHR‐TOF instrument. TEM measurements were performed on a JEOL‐2010 TEM. XRD patterns were collected on a Bruker D4 diffractometer at a scanning rate of 3° min^−1^ in the 2θ range from 10° to 60° (Cu Kα radiation, λ = 1.54056 Å). FTIR spectra were measured in a Magna 750 FTIR spectrometer from samples in KBr pellets. UV‐vis absorption spectra were recorded on a Perkin‐Elmer Lambda 365 UV/Vis/NIR spectrometer. Emission spectra were collected by Edinburgh FLS980 spectrometer. The FL decays of IR808 were induced by a pulsed diode laser (PDL 800‐B) at 797 nm, and recorded by FLS980 spectrometer.

*Synthesis of NaGdF_4_:18%Yb,2%Er Cores*: NaGdF_4_:18%Yb,2%Er cores were synthesized via a modified SLTD method.^12^ A mixture of Gd(CH_3_COO)_3_⋅6H_2_O (0.8 mmol), Yb(CH_3_COO)_3_⋅6H_2_O (0.18 mmol), Er(CH_3_COO)_3_⋅6H_2_O (0.02 mmol), OA (8 mL), and ODE (12 mL) was heated up to 180 °C under N_2_ flow with stirring for 20 min to form a clear homogenous solution. After cooling down to room temperature (RT), NaHF_2_ (2 mmol) was added. Under N_2_ flow, the reactants were heated up to 250 °C with vigorous stirring for 30 min, then increased to 310 °C for another 30 min. After the solution was cooled naturally, the mixture was precipitated by adding ethanol, then centrifuged and washed with ethanol and cyclohexane for three times.

*Synthesis of NaGdF_4_:18%Yb,2%Er@NaGdF_4_:20%Yb UCNPs*: NaGdF_4_:18%Yb,2%Er@NaGdF_4_:20%Yb core–shell UCNPs were synthesized through epitaxial growth with SLTD method. As‐synthesized cores (0.5 mmol), Gd(CH_3_COO)_3_⋅6H_2_O (0.8 mmol), Yb(CH_3_COO)_3_⋅6H_2_O (0.2 mmol), OA (8 mL), and ODE (12 mL) were mixed and heated to 180 °C to form a homogenous solution, and then cooled down to RT. Subsequently, NaHF_2_ (2 mmol) was added to the solution, which was slowly heated to 310 °C and maintained for 25 min under N_2_ protection. After the solution was cooled naturally, core–shell UCNPs were precipitated from the solution with ethanol, washed with cyclohexane and ethanol three times, and finally redisposed in cyclohexane. NaGdF_4_ (NPs) and NaGdF_4_:18%Yb,2%Er@NaGdF_4_:10%Yb,10%Nd (UCNPs@Nd) were also synthesized by the similar procedure except for the different amounts of doping ions. The content of UCNPs, UCNPs@Nd, NPs, and corresponding nano‐composites involved in the subsequent experiments were calculated based on the molar concentration of cores.

*Synthesis of NIR Dye IR808*: IR808 was synthesized in three steps (Scheme S1, Supporting Information). First, 2,3,3‐trimethylindolenine (62 mmol, 6.3 g) and 1,4‐butanesultone (94 mmol, 8.2 mL) were slowly added to o‐DCB (50 mL) in 100 mL round‐bottomed flask and the reaction mixture was kept refluxing at 110 °C for 18 h. The resulting solution was cooled to RT and added dropwise to the ice diethyl ether to precipitate the product. The precipitate was filtered, purified by extraction with H_2_O and chloroform three times, and dried. Second, the precipitate (3 g, 10.2 mmol) and anhydrous sodium acetate (0.836 g, 10.2 mmol) were slowly added to ethanol (150 mL) and the reaction mixture was kept refluxing at 83 °C for 30 min under N_2_ flow. *N*‐[(3‐(anilinomethylene)‐2‐chloro‐1‐cyclohexen‐1‐yl)methylene]aniline hydrochloride (1.832 g, 5.1 mmol) in ethanol (10 mL) was added via syringe. The resulting mixture was refluxed for 18 h and then cooled to RT naturally. Diethyl ether was added to precipitate the product IR783. Finally, IR783 was used to prepare the carboxylic acid‐functionalized derivative IR808 following the literature.^15^ Under an N_2_ atmosphere and dark condition, IR783 (50 mg, 0.06 mmol) and 4‐mercaptobenzoic acid (40 mg, 0.12 mmol) were mixed in DMF (5 mL) at RT for 24 h resulting in a green solution. Solvents were removed by a rotavapor at 60 °C, and the residue was dissolved in dichloromethane (5 mL). Diethyl ether (200 mL) was added slowly to precipitate the product. The as‐obtained IR808 was collected by centrifugation, washed with diethyl ether several times, and dried under 55 °C. ^1^H NMR (400 MHz, d^6^‐DMSO): δ 8.54 (d, *J* = 14.1 Hz, 2H), 7.87 (d, *J* = 8.5 Hz, 2H), 7.53 (d, *J* = 7.4 Hz, 2H), 7.45 (d, *J* = 8.9 Hz, 2H), 7.37 (d, *J* = 1.4 Hz, 2H), 6.40 (d, *J* = 16.4 Hz, 2H), 2.81 (t, *J* = 5.6 Hz, 4H), 1.78 (m, 10H), 1.39 (s, 12H). MS (MALDITOF‐MS): calcd. for C_45_H_51_N_2_O_8_S_3_^+^, 843.2808 [M]^+^; found 843.2798 [M]^+^.

*Synthesis of IR808‐UCNPs*: OA‐capped core–shell UCNPs (0.1 mmol) were subjected to acid treatment to remove the surface ligands,^18^ then mixed with different amount of IR808 (0–0.012 µmoL) in 3 mL of DMF by ultrasonication for 1 min.

*Synthesis of IR808‐UCNPs‐F127*: OA‐capped core–shell UCNPs (0.1 mmol) and different amount of IR808 (0–0.54 µmoL) were dispersed in dichloromethane (10 mL) with vigorous stirring for 1 h. Subsequently, F127 (130 mg) in dichloromethane (4 mL) was added, and the mixture was ultrasound for 2 min at RT. Solvents were removed by a rotavapor and the residue was dissolved in H_2_O. Free IR808 and F127 were removed by centrifugation (14 000 rpm, 4 °C, 20 min). The collected solid was washed repeatedly with H_2_O by centrifugation. The resulting IR808‐UCNPs‐F127 were re‐dispersed in 3 mL water to form a homogenous clear solution. IR808‐UCNPs@Nd‐F127 and IR808‐NPs‐F127 were prepared with the same procedure except for the core–shell UCNPs being replaced with UCNPs@Nd and NPs, respectively. The actual amount of IR808 loaded on nanoparticles was calculated by subtracting the dye content in the supernatant from the total and quantified by UV‐vis absorbance.

*Detection of Hypochlorite In Vitro*: The solutions of IR808‐UCNPs‐F127 or IR808‐NPs‐F127 were reacted with different amount of freshly prepared NaClO solution (0.16 m) under ultrasound for 30 s (or standing for 30 min to reach complete reaction, Figure S14, Supporting Information), then underwent detection. The UCL emission spectra of IR808‐UCNPs‐F127 or IR808‐UCNPs@Nd‐F127 were collected under the excitation at 980 or 808 nm. The emission spectra of IR808‐NPs‐F127 were recorded with a xenon lamp at 760 nm as the excitation source.

*Preparation of Cell Lysates*: HELF cells were lysed by the NP‐40 method according to well‐established protocols. Briefly, cells were trypsinized from culture dishes, washed twice with PBS (pH 7.4), and resuspended in 1.5 mL of PBS at concentration of 1.0 × 10^6^ cells mL^−1^. The cells were lysed in ice bath with Ultrusonic Cell Disrupter System for 15 min, and then the mixture was centrifuged at 16 000 rpm for 20 min at 4 °C to remove any cell debris. The lysates were flash frozen in liquid nitrogen and stored in −80 °C for further use.

*ROS and RNS*: Superoxide (·O^2−^) was prepared from the decomposition of KO_2_. Hydroxyl radical (·OH) was generated through the reaction of H_2_O_2_ and FeSO_4_ at RT. Nitric oxide (NO) was produced by the decomposition of diethylamine NONOate sodium salt hydrates.

*Cytotoxicity of IR808‐UCNPs‐F127*: MCF‐7 cells were provided by Shanghai Institute of Cell Biology, and were grown in culture medium RPMI‐1640 (GIBCO BRL), supplemented with 10% (v/v) heat‐inactivated fetal calf serum, penicillin (100 U mL^−1^), and streptomycin (100 U mL^−1^) at 37 °C under humidified air containing 5% CO_2_. For the cytotoxicity test of NCs, cells were seeded in 96‐well plates with 10^4^ cells per well. In a typical cytotoxicity test of IR808‐UCNPs‐F127, MCF‐7 cells were incubated with fresh medium containing different mass concentrations (0–500 µg mL^−1^) of IR808‐UCNPs‐F127 for 12 or 24 h. After replacing the culture media with cultural media containing CCK‐8 solution, cells were further cultured for 0.5 h and then subjected to absorbance measurement to determine the cell viability by the formula: cell viability (%) = (mean of Abs value of treatment group/mean of Abs value of control) × 100%.

*Intracellular Image and Detection*: MCF‐7 cells were seeded in 20 mm confocal dishes and incubated in RPMI‐1640 containing 0.2 mg mL^−1^ of IR808‐UCNPs‐F127 at 37 °C for 4 h under 5% CO_2_, and then washed with enough PBS to remove excess NPs. Cell imaging was performed on a confocal laser scanning microscope (Nikon Ti‐E&C2) excited by a 980 nm NIR laser. The UCL images were collected in the green channel (500–560 nm) by a photomultiplier tube camera.

For the detection of hypochlorite, the cells incubated with IR808‐UCNPs‐F127 were treated with different concentration of NaClO (0 × 10^−6^, 0.5 × 10^−6^, 1 × 10^−6^ , and 2 × 10^−6^ m) for 1 h. The cells were washed with PBS for three times before detection. An NIR dual‐laser confocal microscope system was developed for ratiometric intracellular detection (Figure 4a). The confocal laser microscope was equipped with two independent excitation sources, 980 and 808 nm continuous‐wave laser diodes (2 W, Changchun New Industries Optoelectronics Tech Co. Ltd.) via dichroic mirror (DM1, DMSP900, Thorlab). An oil‐immersion objective lens (100X/1.49, Apo TIRF) was used to focus the excitation light onto the sample cell. The UCL signal from the sample was collected by the same objective lens, reflected by DM2 (DMSP750B, Thorlabs) and flip mirror (FM), and further detected by spectrometer (Acton SpectraPro SP‐2300) to acquire the full spectra or projected onto a CCD camera to acquire images (Nikon DIGITAL SIGHT DS‐Ri1).

## Conflict of Interest

The authors declare no conflict of interest.

## Supporting information

Supplementary
