# Supplementary material for: A Strategy of NIR Dual‐Excitation Upconversion for Ratiometric Intracellular Detection
Source: Adv Sci (Weinh). 2019 Sep 24;6(22):1901874. doi: 10.1002/advs.201901874 (PMC6864516; doi:10.1002/advs.201901874)
Supplement: Supplementary file 1 — Supplementary [file ADVS-6-1901874-s001.pdf]

Copyright WILEY-VCH Verlag GmbH & Co. KGaA, 69469 Weinheim, Germany, 2019.

ADVANCED SCIENCE
Open Access

Supporting Information

for *Adv. Sci.,* DOI: 10.1002/advs.201901874

A Strategy of NIR Dual-Excitation Upconversion for Ratiometric Intracellular Detection

Jianxi Ke, Shan Lu, Xiaoying Shang, Yan Liu, Hanhan Guo, Wenwu You, Xingjun Li, Jin Xu, Renfu Li, Zhuo Chen, and Xueyuan Chen**

Copyright WILEY-VCH Verlag GmbH & Co. KGaA, 69469 Weinheim, Germany, 2019.

Supporting Information

A Strategy of NIR Dual-Excitation Upconversion for Ratiometric Intracellular Detection

Jianxi Ke, Shan Lu, Xiaoying Shang, Yan Liu, Hanhan Guo, Wenwu You, Xingjun Li, Jin Xu, Renfu Li, Zhuo Chen, and Xueyuan Chen**

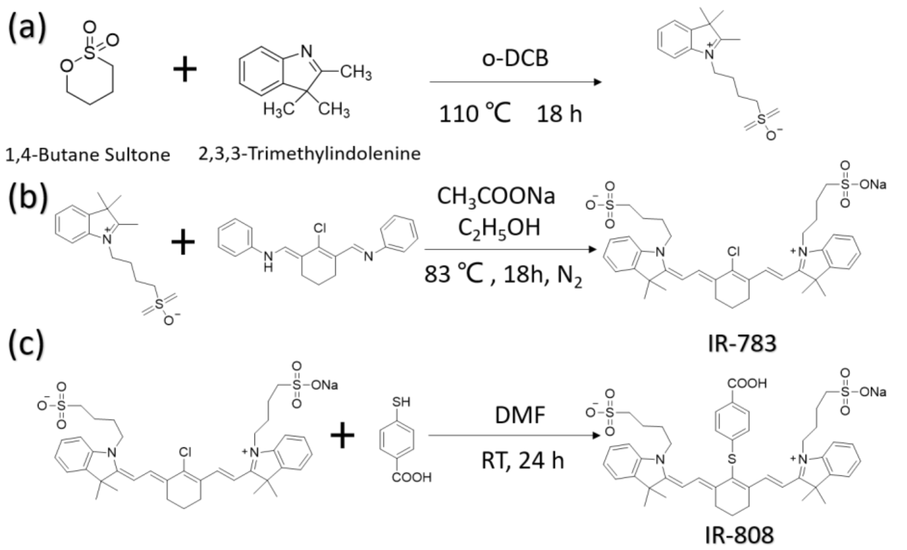

Scheme S1. Synthetic route of the NIR dye IR808.

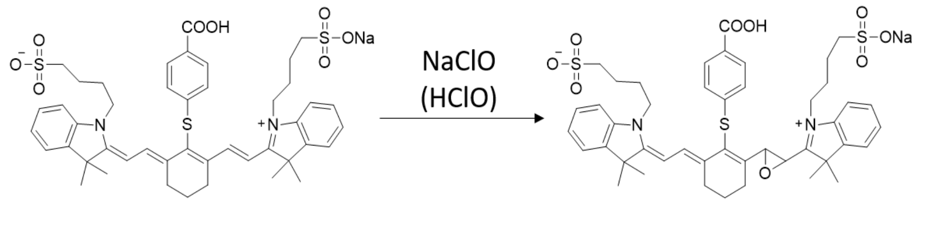

Scheme S2. Possible reaction scheme of IR808 with NaClO. The mechanism was proposed from ^{1}H-NMR spectra, indicative of the conjugated structure changes of IR808.

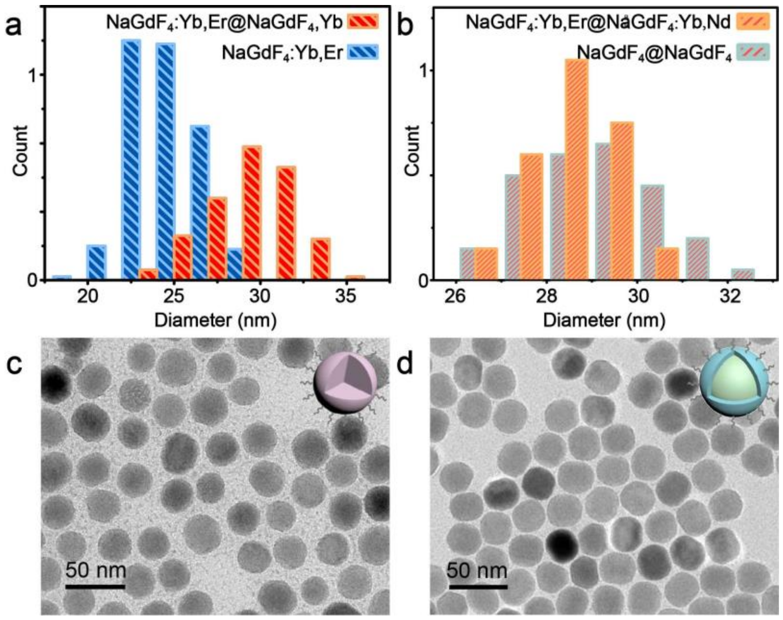

Figure S1. (a) Size distribution histograms of $NaGdF_4$:Yb,Er core and $NaGdF_4$:Yb,Er@$NaGdF_4$:Yb core-shell UCNPs from TEM. (b) TEM images of $NaGdF_4$ (c) and $NaGdF_4$:Yb,Er@$NaGdF_4$:Yb,Nd (d) nanoparticles, and their corresponding size distribution histograms from TEM (b). The mean sizes of $NaGdF_4$:Yb,Er@$NaGdF_4$:Yb,Nd and $NaGdF_4$ nanoparticles were 29.0 and 28.6 nm, respectively, which are similar to that of $NaGdF_4$:Yb,Er@$NaGdF_4$:Yb (29.0 nm).

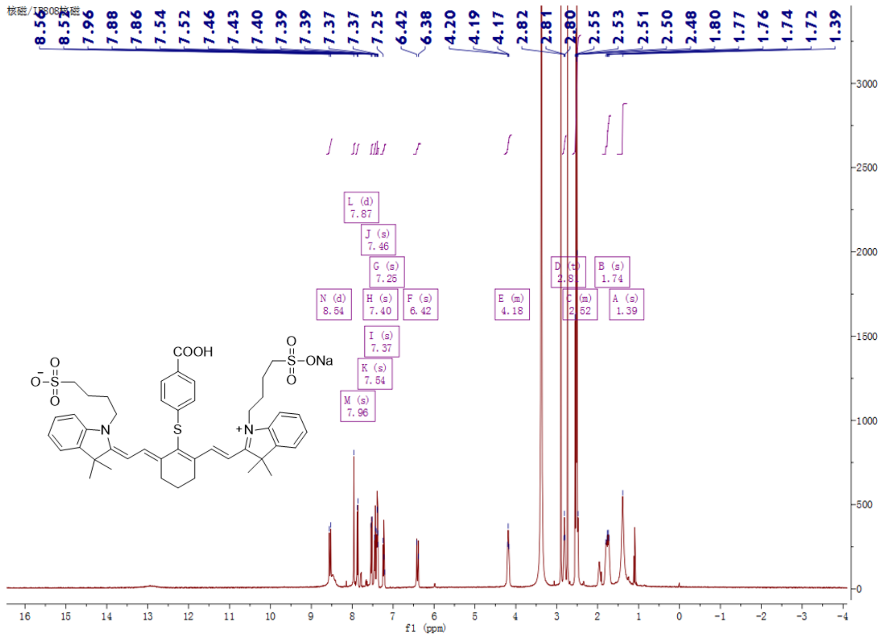

Figure S2. ^{1}H-NMR spectrum of IR808. ^{1}H NMR (400 MHz, d^6-DMSO) δ 8.54 (d, J = 14.1 Hz, 2H), 7.87 (d, J = 8.5 Hz, 2H), 7.53 (d, J = 7.4 Hz, 2H), 7.45 (d, J = 8.9 Hz, 2H), 7.37 (d, J = 1.4 Hz, 2H), 6.40 (d, J = 16.4 Hz, 2H), 2.81 (t, J = 5.6 Hz, 4H), 1.78 (m, 10H), 1.39 (s, 12H).

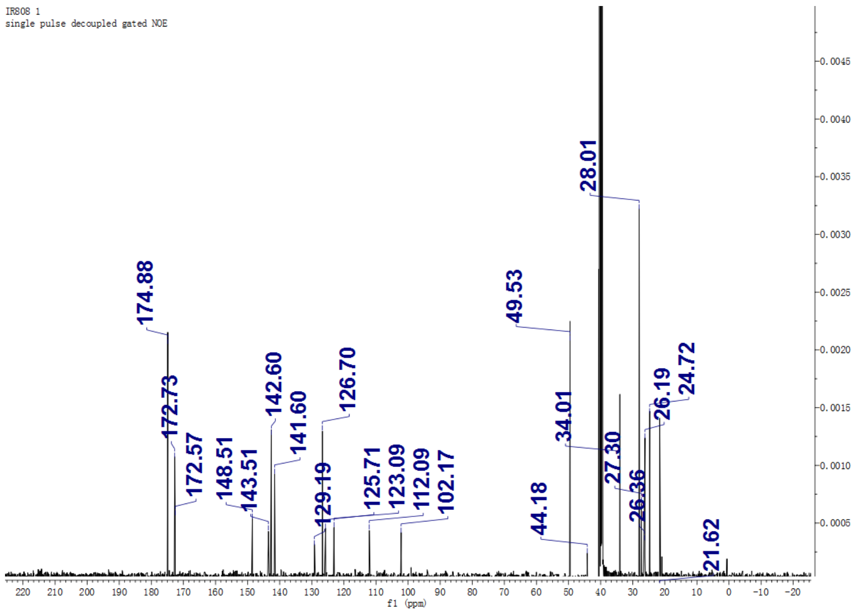

Figure S3. ^{13}C-NMR spectrum of IR808 in d^{6}-DMSO.

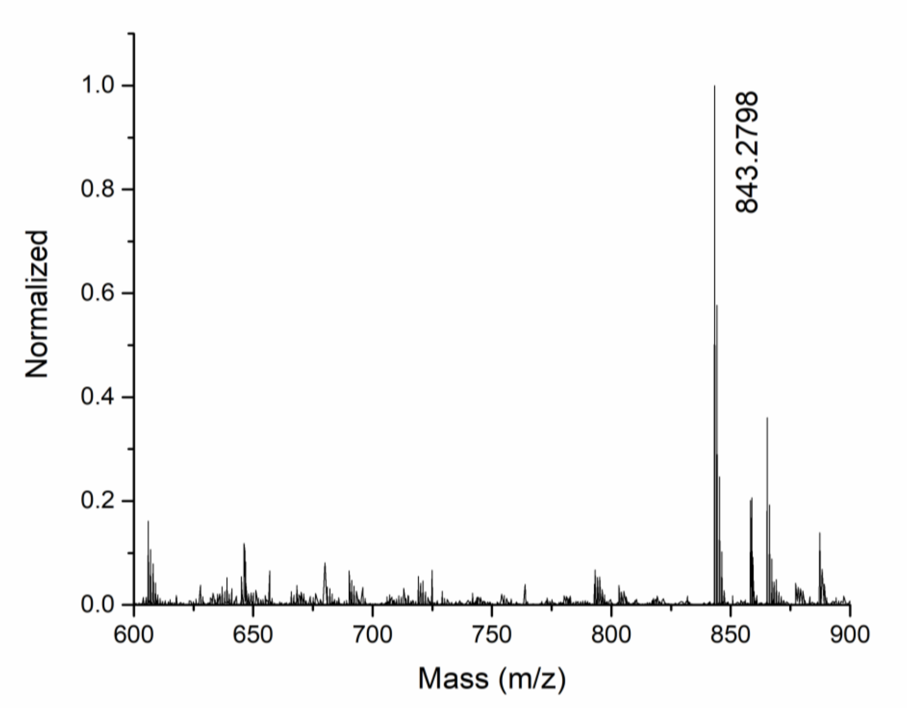

Figure S4. Mass spectrometry (MS) of IR808. MS (MALDITOF-MS): calcd for $C_{45}H_{51}N_2O_8S_3^+$ 843.2808 $[M]^+$; found 843.2798 $[M]^+$.

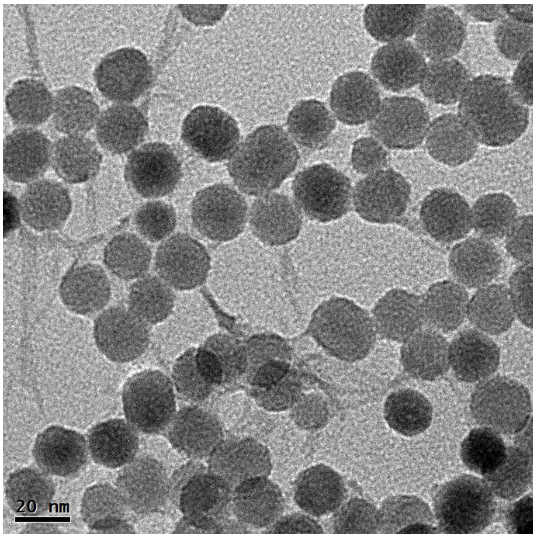

Figure S5. TEM image of IR808-UCNPs-F127, showing IR808-UCNPs-F127 could be well dispersed in water.

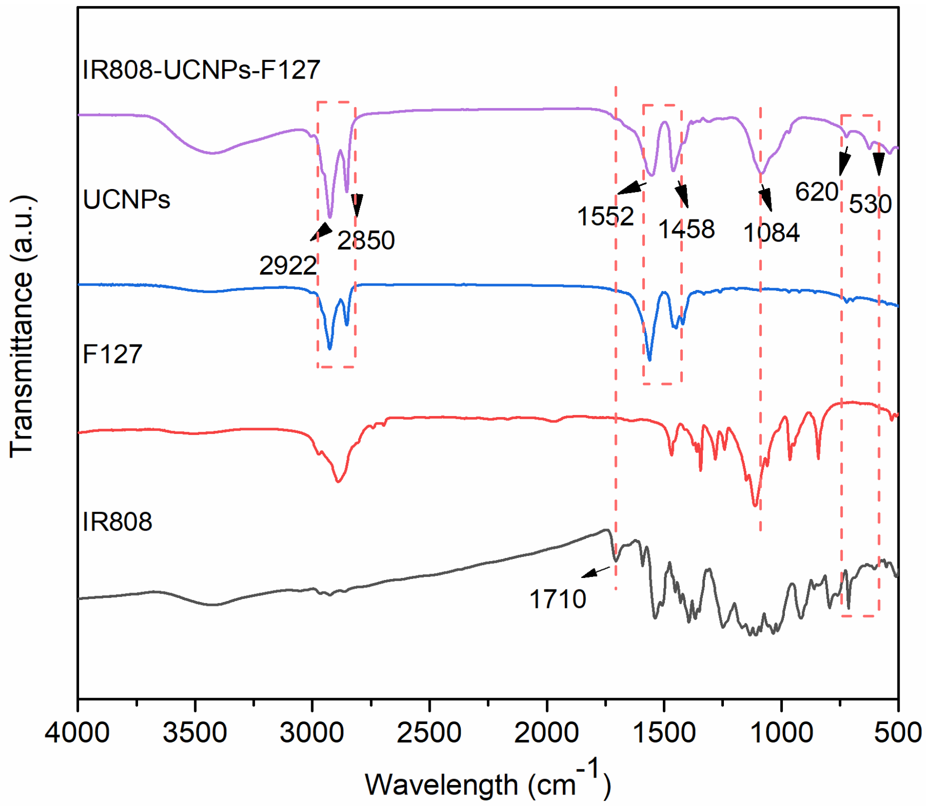

Figure S6. FT-IR spectra of IR808, F127, UCNPs and IR808-UCNPs-F127. The peaks at 2922 and 2850 cm^{-1} were attributed to the asymmetric and symmetric stretching vibrations of methylene ($–CH_2–$) in the long alkyl chain. The bands at 1552 and 1458 cm^{-1} were assigned to the asymmetric and symmetric stretching vibrations of –COO–, indicating that oleic acid (OA) was coated on the surface of the nanoparticles. The peak at 1084 cm^{-1} was ascribed to the C–O–C bond's stretching vibrations in F127 and 620 and 530 cm^{-1} were assigned to the stretching vibration of sulfonic acid group of IR808, confirming the successful assembly of IR808 and F127 on the surface of UCNPs. Furthermore, the C=O stretching vibration mode of the carboxyl group is located at 1710 cm^{-1} for free IR808. After anchoring to the UCNPs, the peak of 1710 cm^{-1} disappeared, indicating that IR808 dye is anchored to UCNPs through carboxyl group.

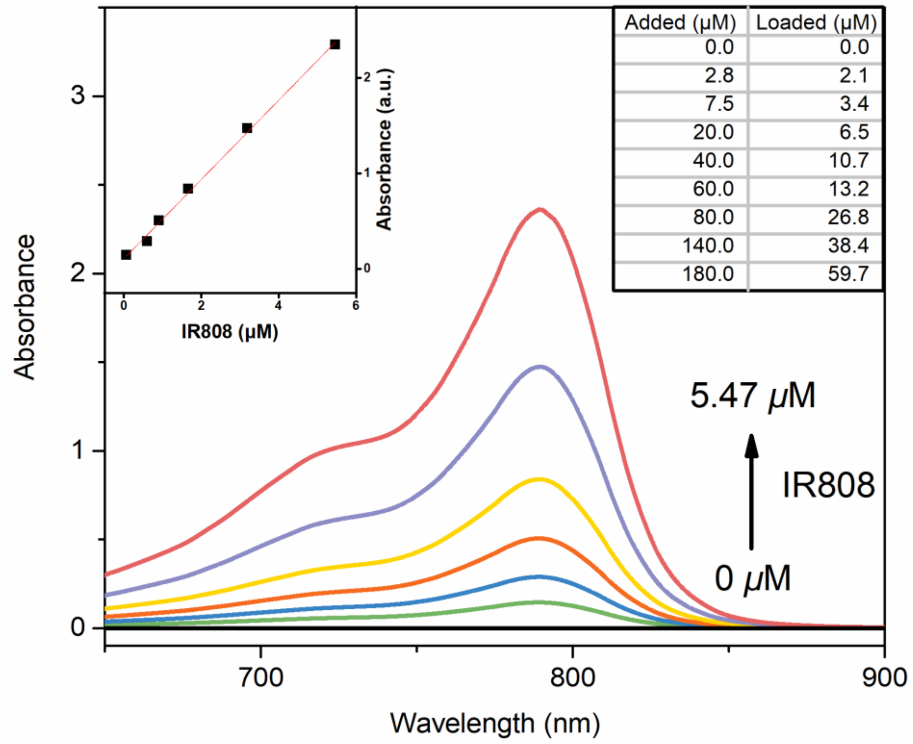

Figure S7. Absorption spectra of IR808 with different concentrations of 0-5.47 μM. Inset: (left) The absorbance at 790 nm as a function of IR808 concentration. (right) The added and loaded dye for preparation of IR808-UCNPs-F127. The amount of IR808 loaded was calculated by subtracting the dye content in the supernatant from the amount added.

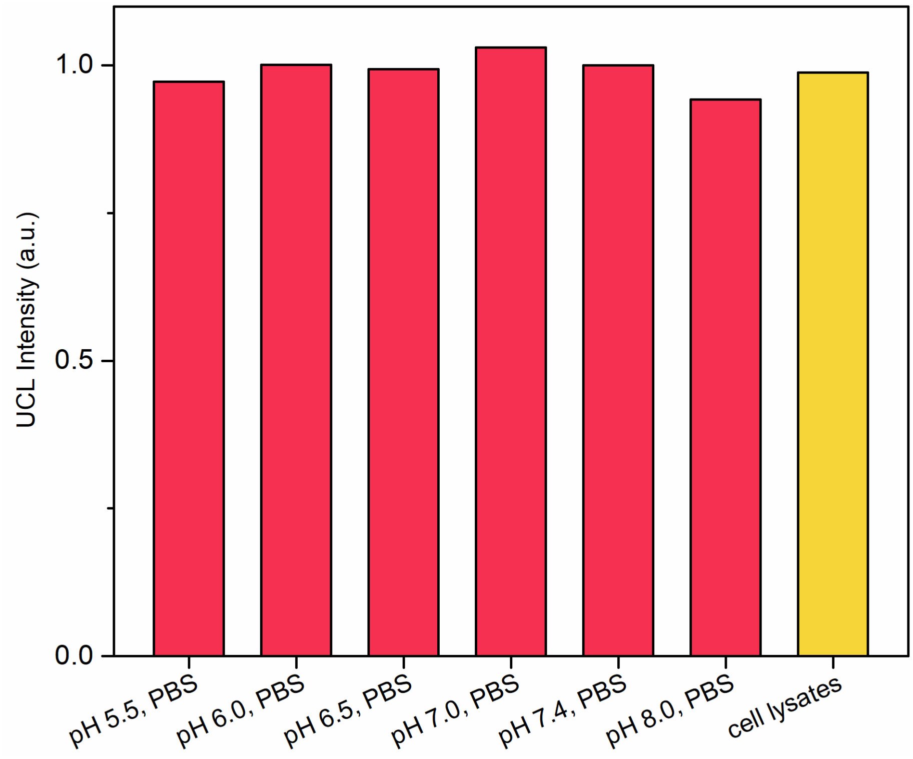

Figure S8. UCL intensity of IR808-UCNPs-F127 nanoprobes in PBS with different pH and the cell lysates of HELF. The nanoprobes were basically stable at physiological pH and intracellular environment.

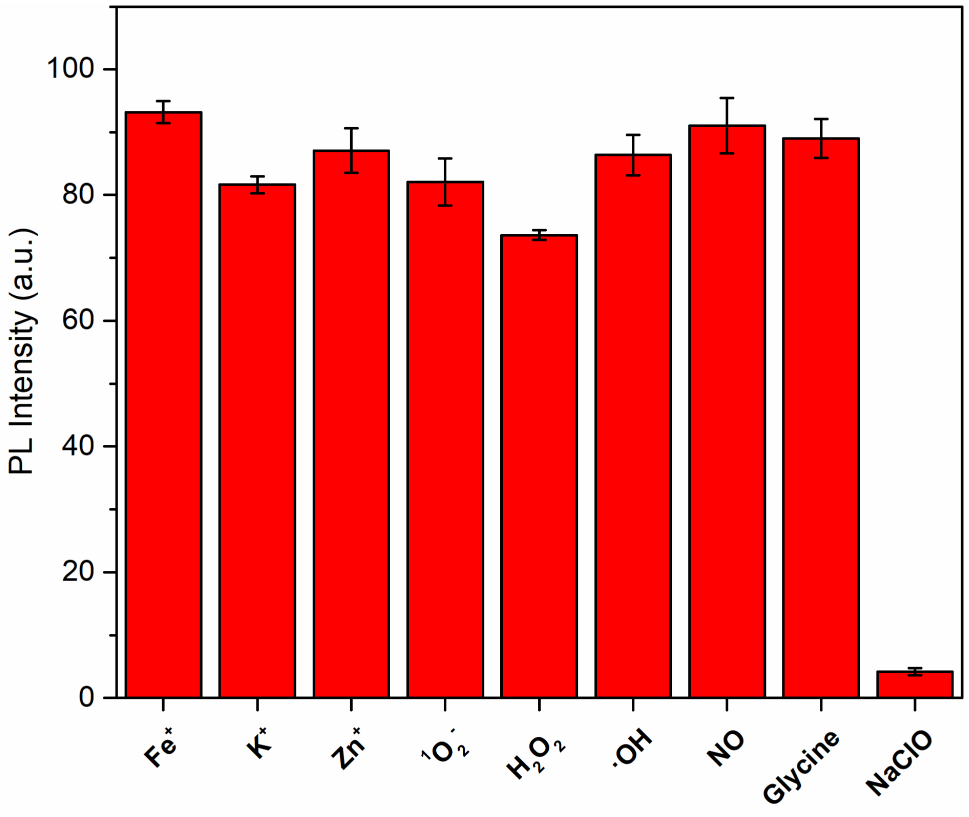

Figure S9. UCL response of IR808-UCNPs-F127 nanoprobes to NaClO (0.4 M) and other biologically relevant analytes (1 M). The nanoprobes showed negligible fluorescence changes in various competing reactive oxygen species (ROS) and other biologically relevant molecules like amino acids and metal ions, indicating that the nanoprobes were highly specific towards NaClO.

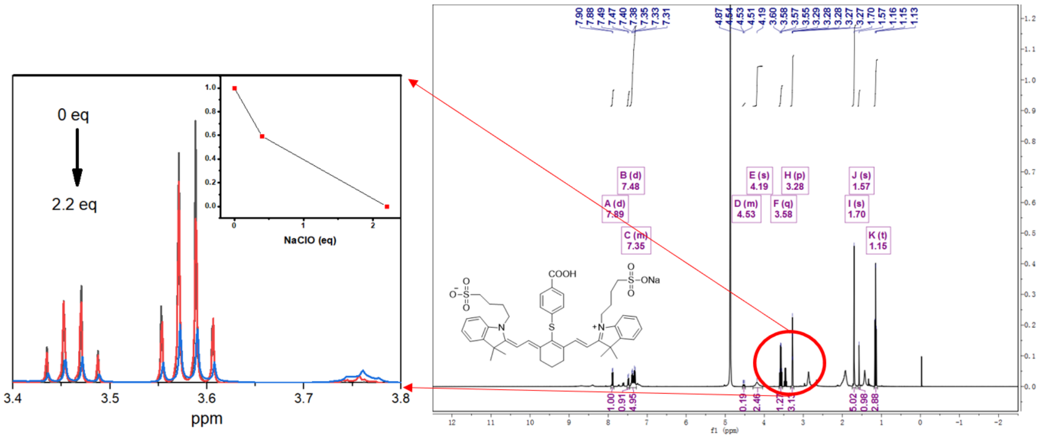

Figure S10. Changes in ^{1}H-NMR spectra of IR808 in Methanol-D4 upon gradual addition of NaClO. The signals (3.4-3.7 ppm) of the C=C double bond in the IR808 were greatly reduced with the treatment of NaClO.

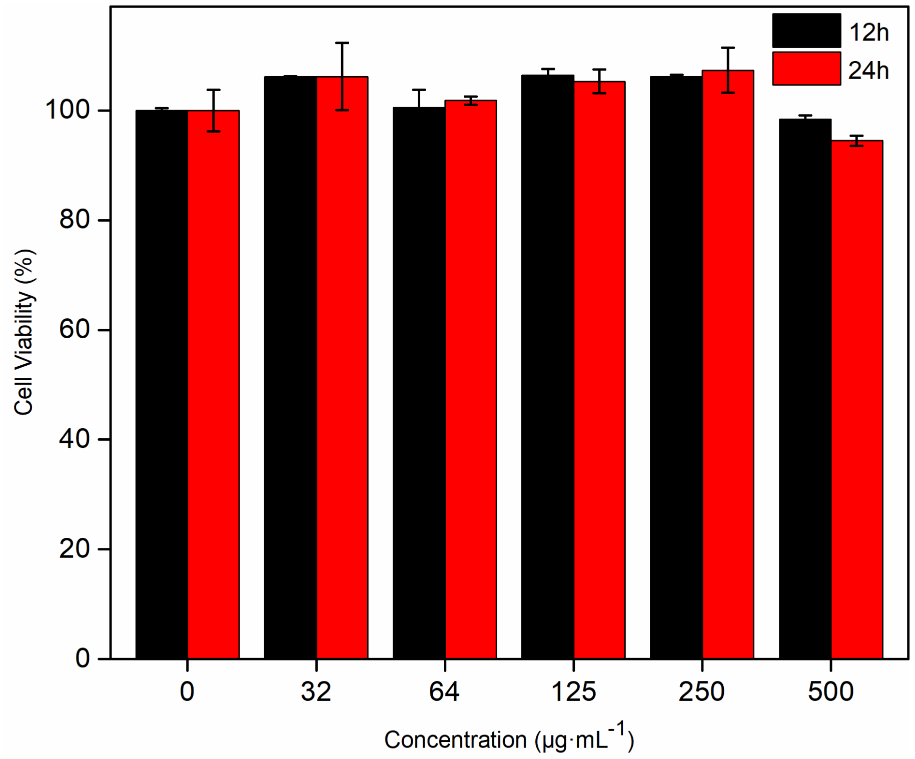

Figure S11. Viability of MCF-7 cells incubated with different concentrations of IR808-UCNPs-F127 nanoprobes for 12 or 24h. All the viabilities of MCF-7 remained above 94%, indicating that the nanoprobes were essentially non-toxic to live cells.

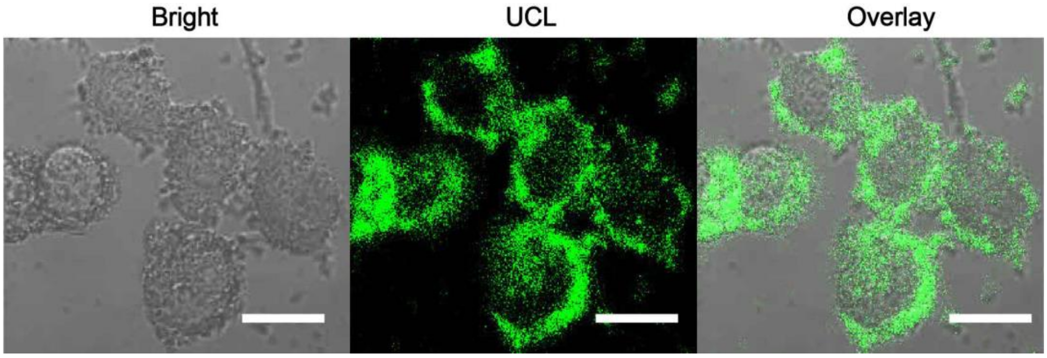

Figure S12. Confocal laser scanning microscopy (CLSM) images of MCF-7 cells incubated with 200 μg·mL^{-1} IR808-UCNPs-F127. Panels 1-3 showed the bright-field image, green UCL image and the overlay image, respectively. The UCL signal was collected by the green channel at 500~560 nm under 980-nm irradiation (scale bar = 10 μM). The images prove the successful cellular uptake of nanoprobes.

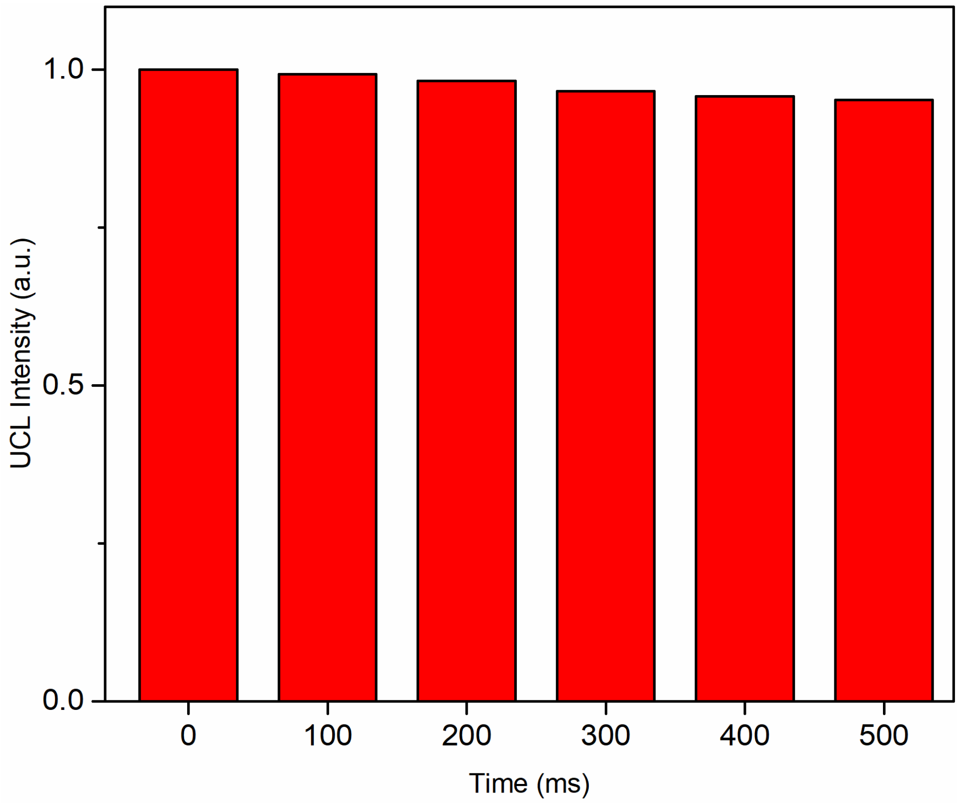

Figure S13. UCL intensities of IR808-UCNPs-F127 for continuous spectral measurements under 808-nm excitation with a power density of ~6×10^4 W/cm^2. The acquisition time for each spectrum was 100 ms. A decline of less than 5% in the UCL intensity was observed after continuous 5 spectral measurements (0.5 s), indicative of reliability of test results.

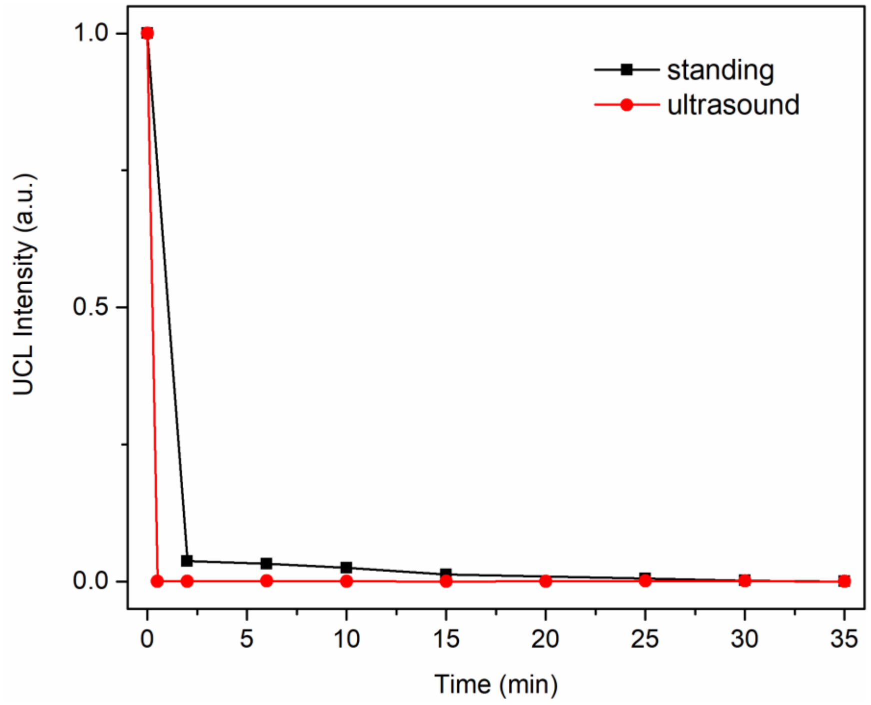

Figure S14. UCL response of IR808-UCNPs-F127 nanoprobes to NaClO (16 mM) at different reaction time by standing or ultrasound. It was found that NaClO induced a strong quenching of UCL, down to less than 5% in 2 min and reached the reaction balance within 30 min. Ultrasound can reduce the reaction time to 30 s. Therefore, for detection of NaClO in vitro, the solution of IR808-UCNPs-F127 was reacted with NaClO by ultrasound for 30 s, then underwent detection. For the intracellular detection, the cells incubated with IR808-UCNPs-F127 were treated with NaClO for 1 h to guaranty a complete reaction.
